# Depression and its impact on health-related quality of life among Chinese inpatients with lung cancer

**DOI:** 10.18632/oncotarget.21001

**Published:** 2017-09-18

**Authors:** Wen Gu, Yan-Min Xu, Jun-Hong Zhu, Bao-Liang Zhong

**Affiliations:** ^1^ Shenzhen Key Laboratory for Psychological Healthcare, Shenzhen Institute of Mental Health, Shenzhen Kangning Hospital, Shenzhen Mental Health Center, Shenzhen, Guangdong Province, China; ^2^ Affiliated Wuhan Mental Health Center, The Ninth Clinical School, Tongji Medical College of Huazhong University of Science and Technology, Wuhan, Hubei Province, China

**Keywords:** depression, lung cancer, quality of life, epidemiology

## Abstract

Depression is of great concern for patients with cancer. A detailed epidemiological profile of depression in Chinese patients with lung cancer and whether depression impacts patients’ health-related quality of life (HRQOL) remain unknown. This study examined the prevalence and socio-demographic and clinical correlates of depression and its effect on HRQOL in Chinese inpatients with lung cancer of two large general hospitals. A total of 148 inpatients were consecutively recruited, and administered with a standardized questionnaire to collect socio-demographic and clinical data. Depression and HRQOL were assessed with the Hospital Anxiety and Depression Scale and World Health Organization QOL Scale Brief Version, respectively. As high as 43.2% Chinese inpatients with lung cancer had clinically significant depressive symptoms. Multiple logistic regression found that depression was significantly associated with moderate-to-severe pain (OR: 4.43), metastatic cancer (OR: 3.63), a short duration after cancer diagnosis (OR: 1.04), poor performance status (OR: 3.41), and small-cell cancer (OR: 4.52). Depressed patients had significantly poorer HRQOL than not depressed patients in terms of all four domains of HRQOL. After controlling for the potential confounding effects of socio-demographic and clinical factors with analysis of covariance, these group-differences in physical (F = 29.074, *P* < 0.001), psychological (F = 76.869, *P* < 0.001), social (F = 21.465, *P* < 0.001), and environmental (F = 27.685, *P* < 0.001) HRQOL remained statistically significant. Depression is prevalent in inpatients with lung cancer and independently associated with poor HRQOL. To address this serious issue, effective pain management, psycho-oncology services and, when necessary, psychiatric assessment and treatment, should be routinely provided in oncology departments of Chinese general hospitals.

## INTRODUCTION

Cancer is the leading cause of mortality in China, with lung cancer being the most common cancer and the leading cause of death due to cancer [1]. Being diagnosed with cancer is a catastrophic event in a person's life, which is particularly devastating for patients with lung cancer due to its short expected survival time [2]. Hopelessness about the future, sadness, pain and suffering, depression, and suicidal behaviors are almost inevitably present. Comorbid depression is a significant concern in patients with cancer, because there is substantial evidence that depression is significantly associated with elevated cancer mortality [3], short survival time [4], elevated risk for suicide [5], and non-adherence to cancer treatment [6].

In China, depression remains largely a hidden issue in clinical oncology practice [7]. For example, in cancer inpatients of a university-affiliated general hospital in Beijing, China, 12.6% met the DSM-IV diagnostic criteria for current major depression, but only 6.9% and 1.7% of the patients with major depression were recognized and referred for psychiatric consultation by their treating oncologists, respectively [8]. Due to the lack of training in clinical psychiatry for Chinese oncologists and other general physicians and inadequate mental health services resources in general healthcare settings, a greater understanding on the epidemiology of depression in patients with cancer might help increase general physicians’ awareness on depression and early identify those at risk for depression.

Studies conducted in Western countries have consistently reported a high prevalence (33–44%) of depressive symptoms in patients with lung cancer, and revealed a variety of significant contributing factors of depression, including gender, age, income, social support, pathological type (i.e., small-cell), performant status, fatigue, and cancer stage [9–14]. In China, only a limited number of studies have investigated the depression of patients with lung cancer [15–20]. Because of varying definitions of depression, various measures of depressive symptoms, and different patient characteristics (i.e., inpatient vs. outpatient), these available studies reported a significantly varying prevalence of depression (15.4–76.3%) [15, 16]. Moreover, nearly all prior studies focused on the most basic socio-demographic correlates of depression among Chinese patients with lung cancer [15–20], a detailed epidemiological profile such as cancer-related factors have not been examined.

Health-related quality of life (HRQOL) is an important outcome measure of chronic illness management and treatment, including anticancer treatment [21, 22]. HRQOL is particularly important for patients with lung cancer, because the survival time of this disease is likely to be short and its treatments are expected to be toxic and limited in efficacy [23]. Studies assessing the HROQL of Chinese patients with lung cancer have observed a poorer HRQOL compared to healthy controls [24–28]. A number of factors, including age, marital status, income, cancer stage, treatment regime, and cell type are found to be significantly associated with poor QOL in Chinese patients with lung cancer [24–28]. Until now, however, the effect of depression on HRQOL of Chinese patients with lung cancer has not been investigated, although there is convincing evidence that depression negatively affects the HRQOL of the general population [29, 30].

Given the clinical significance of depression and its potential association with HRQOL in lung cancer patients, this study was carried out to examine the prevalence and correlates of depression and its impact on HRQOL in Chinese patients with lung cancer.

## RESULTS

The mean age of the 148 participants was 64.8 years (standard deviation [SD]: 11.5, range: 20–99), and 94 (63.5%) were males. In total, 79 patients (53.4%) had metastatic lung cancer, 106 patients (71.6%) were receiving chemotherapy or radiotherapy treatment, and 32 patients (21.6%) had small-cell lung cancer. The average time since the diagnosis of cancer was 24.9 months (SD: 18.4, range: 3–142).

A total of 64 patients had clinically significant depressive symptoms. The prevalence of depression was 43.2% in the whole sample, with 39.4% in men and 50.0% in women. Results of the comparison between not depressed and depressed patients (Table 1) showed that, compared to not depressed patients, depressed patients were significantly more likely to have poor economic status, more intense pain, more advanced-stage cancer, shorter time after cancer diagnosis, a score of 3–4 on the Eastern Cooperative Oncology Group (ECOG) Performance Status Scale, and small-cell lung cancer.

**Table 1:** Characteristics and quality of life (QOL) of lung cancer inpatients with and without depression^*^

Variables	Not depressed inpatients (*n* = 84)	Depressed inpatients (*n* = 64)	Statistics	*P*
Gender: male	27 (42.2%)	37 (57.8%)	χ^2^ = 1.581	0.209
Age (years)	64.6 (11.2)	65.1 (12.1)	t = 0.245	0.807
Education (years)	8.3 (2.6)	7.7 (3.4)	t = 1.211	0.229
Marital status: married or remarried	80 (95.2%)	60 (93.8%)	χ^2^ = 0.157	0.692
Self-rated economic status: poor	23 (27.4%)	31 (48.4%)	χ^2^ = 6.951	0.008
Intensity of pain: moderate or severe	12 (14.3%)	28 (43.8%)	χ^2^ = 15.989	< 0.001
Cancer staging				
Local	24 (28.6%)	7 (10.9%)	χ^2^ = 7.824	0.019
Regional	22 (26.2%)	16 (25.0%)		
Metastatic	38 (45.2%)	41 (64.1%)		
Time since cancer diagnosis (months)	28.5 (21.0)	20.1 (12.9)	t = 3.005	0.003
No. of hospital admissions: > 2	67 (79.8%)	42 (65.6%)	χ^2^ = 3.741	0.053
ECOG Scale score of performance status^#^: 3–4	9 (10.7%)	21 (32.8%)	χ^2^ = 10.976	0.001
Current treatment regimen				
Chemotherapy or radiotherapy	60 (71.4%)	46 (71.9%)	χ^2^ = 0.022	0.989
Palliative care	21 (25.0%)	16 (25.0%)		
Surgery	3 (3.6%)	2 (3.1%)		
Pathological type: small-cell	11 (13.1%)	21 (32.8%)	χ^2^ = 8.333	0.004
WHOQOLBREF				
Physical health	22.2 (3.3)	18.7 (3.3)	t = 6.387	< 0.001
Psychological	18.5 (2.9)	16.6 (3.0)	t = 3.822	< 0.001
Social relationships	10.8 (1.8)	9.3 (1.4)	t = 5.524	< 0.001
Environment	26.5 (4.0)	23.6 (4.1)	t = 4.267	< 0.001

^*^Categorical and continuous variables are expressed as number of cases (%) and mean (standard deviation), respectively. Group-differences in categorical and continuous variables are tested with Chi-square and *t*-test respectively.

^#^1 = Restricted in physically strenuous activity but ambulatory and able to carry out work of a light or sedentary nature, e.g., light house work, office work; 2 = Ambulatory and capable of all selfcare but unable to carry out any work activities; up and about more than 50% of waking hours; 3 = Capable of only limited selfcare; confined to bed or chair more than 50% of waking hours; 4 = Completely disabled; cannot carry on any selfcare; totally confined to bed or chair.

Multiple logistic regression (Table 2) revealed that depression was significantly associated with moderate-to-severe pain (odds ratio [OR]: 4.43), metastatic cancer (OR: 3.63), a short duration after cancer diagnosis (OR: 1.04), poor performance status (ECOG score of 3–4, OR: 3.41), and small-cell cancer (OR: 4.52).

**Table 2:** Multiple binary logistic regression on correlates of depression in inpatients with lung cancer

Factor	Coefficient	Standard error	Wald χ^2^	*P*	OR (95%CI)
Pain intensity: moderate and severe (vs. none and mild)	1.489	0.457	10.635	0.001	4.43 (1.81, 10.85)
Cancer staging: metastatic (vs. local)	1.289	0.565	5.201	0.023	3.63 (1.20,10.98)
Months since cancer diagnosis	0.031	0.015	4.175	0.041	1.04 (1.01,1.07)
ECOG score^#^: 3–4 (vs. 1–2)	1.227	0.515	5.685	0.017	3.41 (1.24,9.35)
Cell type: small (vs. non-small)	1.507	0.514	8.592	0.003	4.52 (1.65,12.35)

^#^1 = Restricted in physically strenuous activity but ambulatory and able to carry out work of a light or sedentary nature, e.g., light house work, office work; 2 = Ambulatory and capable of all selfcare but unable to carry out any work activities; up and about more than 50% of waking hours; 3 = Capable of only limited selfcare; confined to bed or chair more than 50% of waking hours; 4 = Completely disabled; cannot carry on any selfcare; totally confined to bed or chair.

Depressed patients had significant poorer physical, psychological, social, and environmental HRQOL than not depressed patients (Table 1). After controlling for the potential confounding effects of socio-demographic and clinical factors with analysis of covariance (ANCOVA), differences between depressed and not depressed patients remained statistically significant in all domains of HRQOL (physical: F = 29.074, *P* < 0.001, psychological: F = 76.869, *P* < 0.001, social: F = 21.465, *P* < 0.001, and environmental: F = 27.685, *P* < 0.001).

## DISCUSSION

The present study examined the epidemiology of depressive symptoms in Chinese inpatients with lung cancer, as well as their negative impact on the HRQOL of these patients. We found that 43.2% Chinese inpatients with lung cancer in general hospitals experienced clinically significant depressive symptoms in the past week. Compared to two previous studies using the HADS-D, this prevalence is higher than that reported among patients with lung cancer in the United Kingdom (33%) [9], but similar to that reported among a sample of Chinese inpatients with lung cancer from four general hospitals and one cancer specialty hospital in Changsha, China (42.9%) [19]. Our finding that over 40% of inpatients with lung cancer had depression clearly indicates that depression is very common in inpatients with lung cancer in large general hospitals in China.

Overall, our findings on correlates of depression in Chinese inpatients with lung cancer are substantially different from those of previous studies, because we did not identify any significant sociodemographic factors in our analysis, which had been found to be associated with depression of lung cancer patients and the general population [9–20, 31, 32]. Only cancer-related clinical factors were identified as significant correlates of depression in this study. This may be due to the special clinical setting of our study, inpatient departments of large general hospitals, where patients were recently diagnosed with cancer or hospitalized for anticancer treatment, since there is evidence that patients are at particularly high risk for severe emotional distress at the time of disclosure of the cancer diagnosis and treatment [5, 33]. Therefore, cancer-related clinical factors may prevail over or mask other factors in their relationships with depression in inpatient settings.

Evidence from longitudinal studies [34–36] has shown a reciprocal relationship between pain and depression, that is, pain can predict subsequent depression or improving pain results in reduced depression, and vice versa. The significant pain-depression link in our sample might be a reflection of the bidirectional relationship between pain and depression.

The significant association between cancer stage of metastasis and depression is expected, since late-stage cancer is more lethal and less treatable than early-stage cancer [8, 37]. Because patients’ level of psychological distress is the highest at the time when cancer is diagnosed [38], patients with a short duration after lung cancer diagnosis were at higher risk for depression in our study.

Prior studies have found that impaired physical functioning was a risk factor for depression of patients with lung cancer [9, 39]. Consistent with these earlier studies, we found poor performance status was significantly associated with depression. A possible explanations is that patients with difficulties in vital functions (i.e., eating and self-care ability) are more likely to feel hopeless/helpless and thus are at higher risk for depression. In line with an earlier study [9], we found a higher risk of depression in patients with small-cell lung cancer. This may be explained by the unique clinical characteristics of small-cell lung cancer [40], for example, small-cell lung cancer progresses rapidly and cancer cells often have spread within the chest or to other parts of the body when the cancer is diagnosed.

Because depression has many negative effects on physical health, mental wellbeing, social functioning, and even all areas of a person's life [41–43], the significant associations of depression with poor physical, mental, social, and environmental HRQOL were expected. In addition, due to the negative cognition associated with depression and the subjective nature of HRQOL [44, 45], depressed patients may have a negative perception on the environment where they live and work. The negative perception might explain the association between depression and poor environmental HRQOL.

This study has several limitations. First, the study sample of patients with lung cancer were recruited from the inpatient departments of two large general hospitals. Patients from outpatient departments and cancer specialty hospitals were not included. The generalizability of the findings from the present study may be limited. Second, the study was a cross-sectional survey, therefore the causality of associations between depression and clinical variables could not be explored. Due to the same reason, the significant effect of depression on HRQOL is still questionable and needs to be further studied in longitudinal studies. Third, we did not find any significant associations between depression and demographic variables. This may not suggest that sociodemographic variables have no effects on the risk of depression, because the sample size of our study is relatively small and small or moderately sized effects can not be detected due to insufficient statistical power.

In summary, this study has demonstrated a high prevalence of depression in Chinese inpatients with lung cancer, and depression is significantly associated with poor HRQOL in terms of physical health, psychological health, social relationships, and environment. There is a pressing need for health workers of Chinese general hospitals to address the epidemic of depression in oncology departments. Efforts to prevent or reduce depression of patients with lung cancer may be useful to target on those who have pain, metastatic cancer, a short duration after cancer diagnosis, poor performance status, and small-cell cancer. Medical services for patients with lung cancer in oncology departments should include periodic screening for depression, effective pain management, psycho-oncology services, and appropriate psychiatric treatment when necessary.

## MATERIALS AND METHODS

### Subjects

The present study was part of a large-scale cross-sectional survey, which investigated a range of mental health outcomes, suicidal behaviors, and HRQOL among patients with various types of cancer in inpatient departments of two large general hospitals in Tianjin, China, between February and December 2015 [37]. Adult inpatients who were admitted to the two hospitals during the study period, diagnosed with lung cancer (ascertained by histological examination), and had the capacity to provide informed consent, were consecutively invited to participate in the study. We excluded patients who were too ill, had cognitive disorders, or had difficulties in communicating with others. A total of 179 eligible patients were invited and 148 completed the survey.

Before the beginning of the formal survey, this study was approved by the Institutional review Board of Wuhan Mental Health Center and the two participating hospitals. All participants provided written informed consent.

### Procedures and measures

This was a self-administered questionnaire survey. All participants independently and anonymously completed the questionnaires. Trained investigators were assigned to collect cancer-related clinical data by a careful review of medical records and patient interview when necessary.

The primary outcome measure of this study, depressive symptoms, was assessed with the depression subscale of the Hospital Anxiety and Depression Scale (HADS-D) [46]. The HADS-D is composed of seven items. Each item measures a depressive symptom on a 0–3 scale over the prior week, yielding a total score ranging from 0 to 21. Higher total scores indicate more depressive symptoms. A HADS-D score of 9 or higher was used to indicate the presence of clinically significant depressive symptoms for Chinese patients with physical illnesses [47]. The Chinese HADS is reliable and valid for assessing the depressive and anxiety symptoms of physically ill patients [47].

The secondary outcome, HRQOL, was assessed with the Chinese World Health Organization QOL Scale Brief Version (WHOQOL-BREF) [48, 49]. This scale has 26 items, consisting of four domains: physical health, psychological health, social relationships, and environment. Each item is rated on a 5-point Likert scale ranging between 1 (“very dissatisfied/very poor”) and 5 (“very satisfied/very good”). Each domain is scaled in a positive direction with higher scores indicating a better HRQOL.

Sociodemographic variables collected included gender, age, education, marital status, and self-rated economic status (poor, fair, good).

Clinical data included cancer stage (local, regional, metastatic) [50], cell type (small vs. non-small cell), pain intensity, time since the diagnosis of cancer, total number of hospital admissions, functional status, and treatment regime. We used a four-point Verbal Rating Scale to assess the intensity of pain: patients were asked to rate their pain intensity in the last month choosing from the four category responses: 1 = none, 2 = mild, 3 = moderate, and 4 = severe [51]. We used the ECOG Performance Status Scale to evaluate the effect of cancer on patients’ daily living abilities, which is rated on a scale from 0 (fully active) to 5 (dead), with higher score indicating poorer function [52].

### Statistical analysis

Prevalence of depression was calculated. Sociodemographic and clinical characteristics and HRQOL of depressed and not depressed groups were described and compared by *t*-test or Chi-square test, as appropriate. Multivariable logistic regression model with a backward stepwise entry of significant variables in the above univariate analysis was used to identify factors significantly associated with depression. ORs and 95% confidence intervals (CIs) were used to quantify the associations between factors and depression. To determine the independent association of HRQOL with depression, ANCOVA was used to adjust for potential confounding effects of sociodemographic and clinical variables. The statistical significance level was set at P< 0.05 (two-sided). SPSS software version 18.0 package was used for analyses.
